# Differential Gene Expression in Primary Breast Tumors Associated with Lymph Node Metastasis

**DOI:** 10.4061/2011/142763

**Published:** 2011-05-15

**Authors:** Rachel E. Ellsworth, Lori A. Field, Brad Love, Jennifer L. Kane, Jeffrey A. Hooke, Craig D. Shriver

**Affiliations:** ^1^Clinical Breast Care Project, Henry M. Jackson Foundation for the Advancement of Military Medicine, 620 Seventh Street, Windber, PA 15963, USA; ^2^Clinical Breast Care Project, Windber Research Institute, 620 Seventh Street, Windber, PA 15963, USA; ^3^BioReka, LLC, 211 Locknell Road, Timonium, MD 21093, USA; ^4^Clinical Breast Care Project, Walter Reed Army Medical Center, 6900 Georgia Avenue NW, Washington, DC 20307, USA

## Abstract

Lymph node status remains one of the most useful prognostic indicators in breast cancer; however, current methods to assess nodal status disrupt the lymphatic system and may lead to secondary complications. Identification of molecular signatures discriminating lymph node-positive from lymph node-negative primary tumors would allow for stratification of patients requiring surgical assesment of lymph nodes. Primary breast tumors from women with negative (*n* = 41) and positive (*n* = 35) lymph node status matched for possible confounding factors were subjected to laser microdissection and gene expression data generated. Although ANOVA analysis (*P* < .001, fold-change >1.5) revealed 13 differentially expressed genes, hierarchical clustering classified 90% of node-negative but only 66% of node-positive tumors correctly. The inability to derive molecular profiles of metastasis in primary tumors may reflect tumor heterogeneity, paucity of cells within the primary tumor with metastatic potential, influence of the microenvironment, or inherited host susceptibility to metastasis.

## 1. Introduction

Breast cancer is the most common cancer in women from Western countries. In 2009, approximately 190,000 women in the United States were diagnosed with and more than 40,000 died from breast cancer [1]. Progression of malignant breast cancer from localized to systemic disease can lead to impaired organ function, widespread systemic failure, and eventually, death. Five-year survival rates differ dramatically between women with negative lymph nodes (>90%) compared to those with lymph node metastasis (<70%) [2]. Lymph node status is not only the most reliable predictor of survival but is also critical in developing treatment regimens [3]. 

Assessment of lymph node status was originally performed by axillary lymph node dissection (ALND); however, ALND is associated with significant morbidities and has not been associated with significant survival advantage [4, 5], thus alternate methods of evaluating lymph node status have been developed. Sentinel lymph node biopsy (SLNB) assesses lymph node status in the sentinel or first-draining nodes along the axillary lymph node chain; on average, two-three lymph nodes are removed and patients with negative lymph node status are spared complete axillary dissection. Recent results from the NSAPB 32 and ACOSOG Z0011 trials demonstrated that in patients with node-negative disease, SLNB is as effective as ALND, and in patients with positive nodes, despite the risk of axillary recurrence, SLNB performed without follow up ALND is reasonable for patients with early-stage breast cancer [6, 7]. 

 Although SLNB is associated with lower morbidities, surgical disruption of the lymphatic system can result in serious side effects, including numbness, decreased mobility and lymphedema, significantly impacting the quality of life of breast cancer patients. For example, lymphedema can result in pain, decreased functional ability, cosmetic deformities and psychological stress [8] and is estimated to affect 10–20% of breast cancer survivors [9]. In addition, SLNB is associated with a false negative rate of 8–10% [4, 10]. Development of a signature that effectively discriminates patients by lymph node status could stratify patients into those needing surgical evaluation of the lymph nodes for prognostic purposes from those at low risk of metastasis who may be spared possible serious side effects as well as identify those 8–10% of patients misdiagnosed with negative lymph node status after SLNB, who may in fact benefit from more aggressive treatment. In this study, microarray-based gene expression analysis was performed on primary breast tumors from patients with and without metastatic lymph nodes to identify molecular signatures associated with lymph node metastasis. 

## 2. Materials and Methods

### 2.1. Tissue Samples

Tissue samples in the Clinical Breast Care Project (CBCP) tissue bank were collected with approval from the Walter Reed Army Medical Center Human Use Committee and Institutional Review Board. All subjects enrolled in the CBCP voluntarily agreed to participate and gave written informed consent. Clinical information was obtained for all CBCP samples using questionnaires designed by and administered under the auspices of the CBCP. The CBCP database was queried to identify all patients diagnosed with invasive breast cancer between 2001 and 2008. Patients with a previous history of breast cancer, documented BRCA1 or BRCA2 mutations, or who underwent neoadjuvant therapy were not eligible for this study. Patients with isolated tumor cells or micrometastases as well as those diagnosed with negative lymph node status who later died of disease were excluded from analysis. To ensure consistency, diagnosis of every specimen was made by a single breast pathologist from hematoxylin and eosin (H&E) stained slides; grade was assigned using the Nottingham Histologic Score [11, 12]. ER and PR status were determined by immunohistochemistry by a commercial clinical laboratory (MDR Global, LLC, Windber, PA, USA); HER2 status was determined by fluorescence in situ hybridization using the PathVysion HER2 kit according to manufacturer's protocol (Abbott Laboratories, Abbott Park, IL, USA). 

### 2.2. RNA Isolation, Amplification, aRNA Labeling and Hybridization

For each case, hematoxylin- and eosin-stained slides were examined by a dedicated breast pathologist and tumor areas marked for laser microdissection. One to six serial sections (8 *μ*m thick) were cut, mounted on glass PEN foil slides (W. Nuhsbaum, Inc., McHenry, IL, USA), stained using the LCM staining kit (Applied Biosystems, Foster City, CA, USA) and microdissected on an AS*LMD* laser microdissection system (Leica Microsystems, Wetzlar, Germany). Slide preparation, staining and cutting were performed within 15 minutes to preserve RNA integrity. 

 RNA was isolated from laser microdissected tumor cells using the RNAqueous-Micro kit (Applied Biosystems, Foster City, CA, USA) and treated with DNase I to remove any contaminating genomic DNA. RNA integrity was assessed using the 2100 Bioanalyzer (Agilent Technologies, Santa Clara, CA, USA). RNA was converted to biotin-labeled aRNA using two rounds of amplification with the MessageAmpII aRNA Amplification kit (Applied Biosystems, Foster City, CA, USA), and the concentration and quality of the aRNA samples measured with the NanoDrop ND-1000 (NanoDrop Products, Wilmington, DE, USA) and the 2100 Bioanalyzer, respectively. Hybridization, washing, staining and scanning were performed using the HG U133A 2.0 arrays (Affymetrix, Santa Clara, CA, USA) according to manufacturer's protocol. 

### 2.3. Analysis and Statistics

Affymetrix gene expression data was imported into Partek Genomics Suite 6.5 (Partek, Inc, St Louis, MO, USA) as CEL files using default Partek parameters. Raw data was preprocessed, including background correction, normalization and summarization using robust multiarray average (RMA) analysis and expression data log2 transformed. Differential gene expression analysis was performed using one-way ANOVA using lymph node status as the variable. Gene lists were created using a cut-off of *P* < .001, >1.5-fold change. Hierarchical clustering was performed using the Gene Expression module. 

## 3. Results

### 3.1. Clinicopathological Characteristics

The average number of metastatic lymph nodes in the node-positive group was 4.69 (range 1–19). The average age at diagnosis did not differ significantly between those with (55.0 years) and those without (57.7 years) lymph node metastasis. None of the pathological features evaluated differed significantly between groups (Table 1). To date, 24/39 (62%) of the node-negative patients have been disease-free for ≥5 years and none have died of disease. In contrast, 6/35 (17%) of node-positive patients died of disease with an average survival of 34 months, one (3%) has progressed from stage IIIa to stage IV, and 47% have remained disease-free for at least five years.

### 3.2. Gene Expression

**Figure 1:**
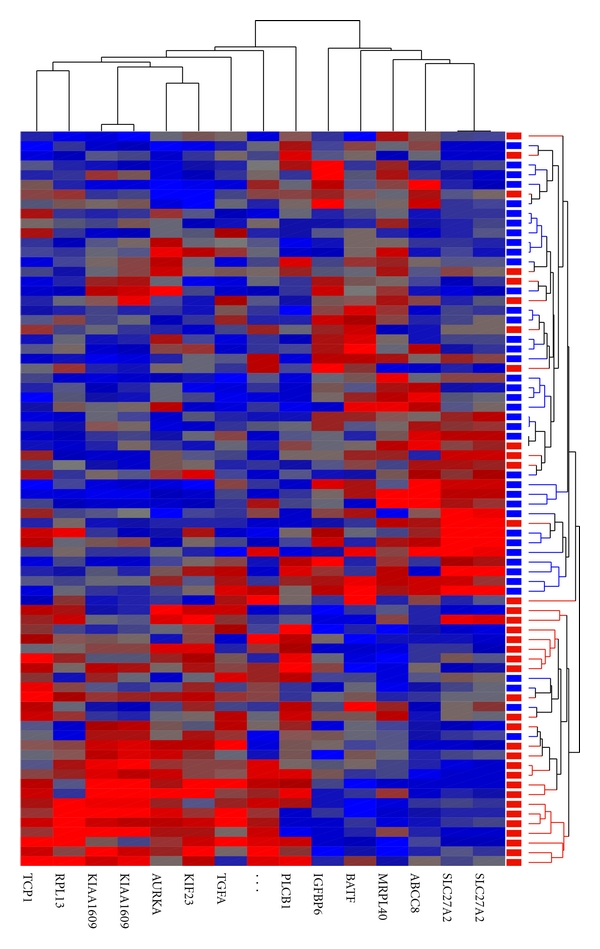
Heat map and hierarchical clustering of 76 primary tumor samples based on 15 differentially expressed probes. Tumors from patients with negative lymph node status are represented in the dendrogram by blue bars and tumors from patients with positive lymph node status are represented by red bars. 4/41 tumors with negative lymph nodes and 12/35 tumors with positive lymph nodes were classified incorrectly. Red squares: high expression, blue: low expression.

Statistical analysis revealed significant differences in expression levels for 15 probes between tumors from patients with lymph node metastases and those without (Table 2). These genes correspond to 11 genes (KIAA1609 and SLC27A2 are each represented by two independent probes) with known function, one uncharacterized gene and one probe that represents a UniGene EST cluster only. These results suggest that primary breast tumors with different metastatic capacities are more similar than different in gene expression as the small number of genes differentially expressed does not differ significantly (*P* = .25) from what would be expected by chance. Hierarchical clustering analysis was able to correctly classify 4/41 (90%) of the lymph node-negative tumors but only 23/35 (66%) of the node-positive tumors (Figure 1).

## 4. Discussion

Gene expression-based molecular signatures have been developed that can be used to predict intrinsic subtype, tumor grade, and risk of recurrence [13–15], each of which can be used as a prognostic tool. Although a signature specific to the development of local metastases may not predict overall outcome, such a signature would have both biological and clinical utility. Identification of genes involved in the successful establishment of metastatic tumors in the lymph nodes would improve our understanding of the metastatic process. Differentially expressed genes may represent those involved in the initiation of metastasis, altering cell motility, angiogenesis and invasion thus allowing primary tumor cells with metastatic potential to disseminate [16]. These genes would then serve as molecular targets against which novel therapeutics could be developed to prevent the early stages of metastasis. In addition, identification of a signature of metastasis would allow women at low risk of lymph node metastasis to be spared unnecessary surgical procedures and the ensuring complications of lymph node disruption as well as to identify the 8–10% of node-positive women diagnosed as node-negative by SLNB [17]. 

To this end, efforts have been made to develop a breast tumor molecular signature that differs between patients with and without lymph node metastasis. For example, evaluation of gene expression patterns of 176 candidate genes between primary tumors without lymph node metastasis and those with 10 or more positive lymph nodes revealed differences in gene expression, with significantly higher expression of ERBB2 (*P* < .0001) in tumors from node-positive compared to node-negative tumors [18]. From a pool of 89 primary tumors, data from 19 primary tumors without lymph node metastasis and 18 with ≥10 positive lymph nodes were compared to generate a metagene profile, enriched for genes involved in cellular immunity, capable of predicting lymph node status with 90% accuracy [19]. Finally, using Serial Analysis of Gene Expression in 27 invasive ductal carcinomas with either positive or negative lymph node status, 245 differentially expressed (*P* < .05) genes were detected; these results were validated in an independent set of tumors for seven of the genes [20]. 

In contrast, a number of research groups have failed to develop molecular signatures predictive of lymph node metastasis. Gene expression data from 129 primary breast tumors was used to successfully develop signatures correlating expression patterns with grade and ER and HER2 status but a signature for lymph node status could not be identified; the authors thus concluded that while there may be a biological propensity to metastasize, the influence of time and stochastic processes on tumor metastasis may preclude the identification of a signature of lymph node metastasis [21]. In a second study evaluating microarray data from 151 lymph node-negative and 144 lymph node-positive primary tumors, significant gene expression differences were not detected between tumor types. The authors then applied the lymph node metastasis signature described previously by Huang et al. to their own external data set and achieved a classification accuracy of only 50%, implying that the signature developed by Huang, using a small sample set and limiting analysis to patients with ≥10 positive lymph nodes, is not an effective predictor of nodal metastasis [22]. In addition, while the 70-gene poor prognosis signature that is the basis for the MammaPrint assay is effective at predicting risk of recurrence, it was ineffective in predicting lymph node status, leading the authors to conclude that hematogenous and lymphogenic metastases are driven by independent molecular mechanisms [23]. Similar to these studies, the fifteen probes found in our study to be differentially expressed were not effective in correctly classifying primary tumors, especially those with positive lymph nodes, by lymph node status. 

A number of reasons may explain the discrepancy between those groups that have reported molecular signatures of lymph node metastasis and those that have failed to find gene expression differences. Study design may affect the ability to detect critical molecular alterations. Most studies identifying a signature of lymph node metastasis relied on small (<40 samples) sample sizes. Breast cancer is not a single disease but rather a complex mix of different architectures, grades and underlying subtypes which may necessitate the use of large number of samples to generate robust signatures [24]. In addition, multiple models were developed using patients with extremely discordant (negative lymph node status compared to ≥10 positive lymph nodes) phenotypes [18, 19]; these models, therefore, may not apply to the majority of patients who have an intermediate number of positive lymph nodes [21]. Finally, validation of these signatures on independent sample sets has, to our knowledge, not been reported, and to date, while molecular portraits are used to determine tumor grade, subtype and prognosis, no clinical assay is available to determine lymph node status. 

In addition to methodological concerns, lack of a signature of lymph node metastasis may be attributable to biological properties of primary breast tumors, such as the nature and number of cells within a primary tumor with metastatic potential. Injection of melanoma cells into mice demonstrated that tumor cells vary widely in their ability to produce metastases, and cells with metastatic potential are rare within the primary tumor [25, 26]. This view was challenged by the development of gene expression signatures such as the 70-gene poor prognosis signature and a molecular signature of metastasis developed from solid tumors [15, 27]; because these signatures were derived from bulk tumors, the authors concluded that the majority of cells in the primary tumor have the ability to metastasize. In fact, the ability to predict which tumors will metastasize based on gene signatures derived from primary tumors does not preclude the presence of small subpopulations of cells with full metastatic potential found in localized regions throughout the primary tumor [28, 29]. For example, comparison of gene expression patterns between cell line populations that have high compared to low metastatic potential to bone revealed that only a small fraction of cells demonstrated the full bone metastasis signature [30]. More recently, the sequencing of a basal-like primary breast tumor and corresponding brain metastasis revealed a significant enrichment of 20 mutations in the metastasis compared to the primary tumor, suggesting that metastases arise from a minority population of cells within the primary tumor [31]. If these models in which few cells within the primary tumor have full metastatic capacity are correct, genetic signatures from these rare cells will be masked by the majority of tumor cells which do not have full metastatic capacity. 

Molecular heterogeneity within tumor subtypes may also preclude the identification of a single signature of metastasis. Breast tumors can be classified by their intrinsic subtypes, including luminal A, luminal B, HER2-positive and basal-like, based on different patterns of gene expression [13]. These subtypes have been associated with differences in relapse-free and overall survival with the basal-like and HER2-positive subtypes having the shortest survival times [32]. Not only do intrinsic subtypes have different prognoses, but recent studies have shown that each subtype has preferential sites of metastasis: bone was the predominant site of relapse in luminal and HER2-positive tumors but was infrequent in basal-like tumors. In contrast, basal-like tumors had frequent relapse in brain, lung and distant lymph nodes [33, 34]. Data supporting the idea that tumors with different phenotypes may metastasize differently was provided by a recent study which found nonoverlapping signatures for the development of distant metastasis in lymph-node-negative ER-positive and ER-negative tumors, suggesting that there are different molecular mechanisms associated with metastasis depending on tumor biology [35]. Whether lymph node metastasis is similarly affected by tumor phenotypes such as ER status or intrinsic subtype remains to be determined. 

The ability to metastasize may be influenced by not only the tumor cells but also the microenvironment, both local and distant. Dissemination of tumor cells from the primary site is one of the earliest steps of metastasis; successful invasion and migration of tumor cells requires a number of changes in the breast microenvironment including degradation of the extracellular matrix and angiogenesis. Distant tissue may be subjected to premetastatic niche conditioning, undergoing changes such as recruitment of bone-marrow derived cells that form a favorable environment for tumor cells to grow. Finally, the last stages of metastasis require tumors cells to successfully reach the secondary site, escape senescence and survive and proliferate within a foreign environment [16, 36]. Given the importance of the microenvironment, molecular characterization of the tumor component alone may not be sufficient in predicting metastatic behavior as a tumor with an aggressive profile may be growing within a nonpermissive microenvironment and vice versa. In fact, many signatures of poor prognosis or metastasis include the expression of stromal genes. Thus, consideration of only the tumor epithelial component may fail to capture the full metastatic potential of a primary tumor. 

 Finally, the ability to metastasize may depend not on biologic features of the primary tumor but on inherent host susceptibility. Outcrossing of a highly metastatic transgenic mouse to a variety of inbred mouse strains resulted in significant variability in the propensity to metastasize; since each animal received the metastatic transgene, the differences in metastatic capacity have been attributed to genetic background [37]. Linkage studies identified candidate metastasis modifier genes in mouse, including SipaI [38]. Follow up studies in humans confirmed that SIPA1 is a metastasis susceptibility gene [39, 40]. Thus, the ability to successfully metastasize may, at least in part, reflect a systemic, rather than tumor-driven, proclivity.

## 5. Conclusions

New molecular tools are needed that can effectively discriminate patients with and without the propensity to develop lymph node metastasis so that women at low risk may be spared potentially significant morbidities associated with surgical evaluation, and the false negative rate associated with SLNB can be reduced. In this study, 15 probes, representing 11 well-characterized and two hypothetical genes, were differentially expressed between tumor types; however, hierarchical clustering based on this gene signature was ineffective, especially for the lymph node-positive tumors, suggesting that a single molecular classifier for lymph node metastasis may not exist. The inability to derive molecular profiles of metastasis in primary tumors may reflect tumor heterogeneity, paucity of cells within the primary tumor with metastatic potential, influence of the microenvironment, or inherited host susceptibility to metastasis. 

## Figures and Tables

**Table 1:** Clinical and pathological features of 76 invasive breast tumor specimens used in microarray analysis.

	Node negative (*n* = 41)	Node positive (*n* = 35)	P node− versus node+
Age			
<50 years	37%	34%	NS
≥50 years	63%	66%	
Histology			
IDCA	95%	86%	
ILCA	5%	9%	NS
Mixed	0%	5%	
Grade			
Well-differentiated	27%	9%	
Moderately-differentiated	29%	43%	NS
Poorly-differentiated	44%	48%	
Hormone receptor status^a^			
ER+/PR+	54%	51%	
ER+/PR−	22%	12%	NS
ER−/PR−	24%	37%	
HER2 Status			
Positive	20%	26%	NS
Negative	80%	74%	
Tumor Size			
T1	63%	42%	
T2	34%	54%	NS
T3	3%	4%	

^a^No cases of ER−/PR+ were identified in this group of tumors.

**Table 2:** Fifteen probes demonstrating significant differences in expression level between tumors with and without lymph node metastases. KIAA1609 and SLC27A2 were represented by multiple probes.

Gene symbol	Accession number	Gene name	Probe ID	*P* value	Fold-change
Genes downregulated in node-positive primary tumors					

ABCC8	NM_000352	ATP-binding cassette, subfamily C (CFTR/MRP), member 8	210246_s_at	.000889	1.67
BATF	NM_006399	Basic leucine zipper transcription factor, ATF-like	205965_at	.000874	1.51
IGFBP6	NM_002178	Insulin-like growth factor binding protein 6	203851_at	.000679	1.55
MRPL40	NM_003776	Mitochondrial ribosomal protein L40	203152_at	2.14E-05	1.57
SLC27A2	NM_003645	Solute carrier family 27 (fatty acid transporter), member 2	205768_s_at	.000413	2.34
			205769_at	.000921	2.12

Genes upregulated in node-positive primary tumors					

—^a^	AL050145		215526_at	.000477	1.55
AURKA	NM_198433	Aurora kinase A	208079_s_at	.000512	1.80
KIAA1609	NM_020947	KIAA1609	221843_s_at	1.25E-05	1.66
			65438_at	1.53E-05	1.73
KIF23	NM_138555	Kinesin family member 23	204709_s_at	.000675	1.82
PLCB1	NM_015192	Phospholipase C, beta 1 (phosphoinositide-specific)	213222_at	.000612	2.05
RPL13	NM_033251	Ribosomal protein L13	214976_at	.000129	1.64
TCP1	NM_030752	T-complex 1	208778_s_at	.000183	1.51
TGFA	NM_003236// NM_001099691^b^	Transforming growth factor, alpha	205016_at	.000233	1.77

^a^This probe corresponds to UniGene cluster HS.225986 but not to a known gene.

^b^This probe represents both isoforms 1 and 2 of the TGFA gene.
